# Primary adenoid cystic carcinoma of trachea presenting as midline neck swelling and mimicking thyroid tumor: A case report and review of literature

**DOI:** 10.4103/0970-2113.68330

**Published:** 2010

**Authors:** Paras Nuwal, Ramakant Dixit, Anand K. Singhal

**Affiliations:** *Department of Pathology, JLN Medical College, Ajmer, India*; 1 *Department of Respiratory Medicine, JLN Medical College, Ajmer, India*; 2 *Department of ENT, JLN Medical College, Ajmer, India*

**Keywords:** Adenoid cystic carcinoma trachea, thyroid tumor, midline swelling neck

## Abstract

We report an extremely rare case of primary adenoid cystic carcinoma (ACC) trachea presenting as midline swelling neck and mimicking thyroid tumor. A 44-year-old female presented with painless midline swelling neck without any respiratory complaints, hoarseness of voice or dysphagia etc. Fine needle aspiration cytology (FNAC) from swelling reveal features of papillary carcinoma thyroid. Subsequently the operative findings, bronchoscopy and histological diagnosis of excised mass, along with review of FNAC, revealed features of ACC of trachea with exra tracheal extension anteriorly into the soft tissue neck, without actual invasion of the thyroid gland. The world literature on extension of an ACC arising in the laryngotracheal complex to thyroid or soft tissue neck and clinical manifestation as a thyroid nodule or mass is reviewed. The cytological differential diagnosis of ACC and CT findings are also briefly discussed.

## INTRODUCTION

Primary tracheal tumors are rare and constitute only two per cent of all respiratory tract tumors.[1] Adenoid cystic carcinoma (ACC) is a rare primary tracheal tumor that is second most common tracheal malignancy at histology after squamous cell carcinoma.[2]

Most patients with ACC trachea are symptomatic at the time of presentation and symptoms usually relate to airway obstruction i.e. cough, hoarseness, hemoptysis, shortness of breath and wheezing respiration. Patients may be incorrectly diagnosed and treated for asthma or chronic bronchitis for months or years before the lesion is recognized.[3] Very rarely, a tumor arising in the cervical trachea may extend anteriorly and presents as thyroid tumor.[4–8] We present a rare case of primary ACC of trachea presenting as midline swelling neck, mimicking thyroid tumor, without actually invading the thyroid gland. Such a presentation has not been reported in the literature from South East Asian countries previously.

## CASE REPORT

A 44-year-old woman presented with swelling in neck of six months duration. She denied history of cough, breathlessness, chest pain, hemoptysis, dysphagia and hoarseness of voice. Local examination revealed a 2.5 cm × 2.0 cm firm rounded nontender swelling fixed to the underlying structures. It was slightly moving with deglutition. Other systemic examination was unremarkable.

**Figure 1:**
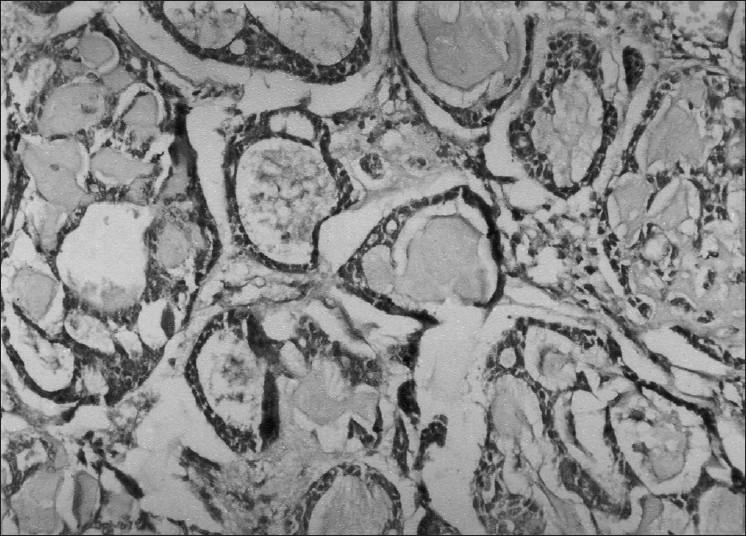
Photomicrograph showing histological features of adenoid cystic carcinoma with uniform hyper chromatic basaloid cells surrounding acellular spaces containing mucoid and hyaline material (H and E, ×100)

**Figure 2:**
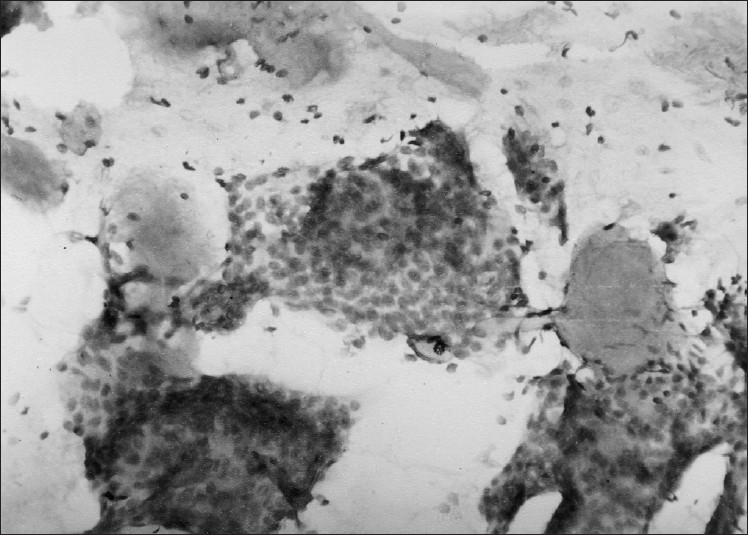
FNAC smear showing spherical globules of basement membrane material and hyper chromatic uniform rounded tumor cells with scanty cytoplasm (May Grunwald Giemsa stain, ×400)

Her investigations revealed normal hemogram and organ functions. X-ray chest was also normal. Patient was referred for FNAC. Cytological findings revealed presence of papillae and acini made of uniform rounded tumor cells with pinkish background suggestive of papillary carcinoma thyroid. The thyroid profile (T_3_, T_4_ and TSH level) was normal. Computed tomographic (CT) scan of the neck and thyroid scan could not be done due to financial and resource constraints. Case was then operated upon and during surgery, it was found difficult to remove the entire mass as it had penetrated deeper soft tissue and was firmly adherent to the trachea. Subtotal thyroidectomy was performed and biopsy specimen was taken from tumor mass. Histopathological examination of the mass revealed uniform basaloid cells arranged in strands or clumps surrounding accelular spaces containing mucoid or hyaline material imparting a ‘Swiss cheese appearance’ to the tumor. An intervening fine delicate connective tissue stroma was also seen. A diagnosis of ACC with cribriform pattern was made [Figure 1]. Sections from thyroid tissue did not reveal any abnormality or tumor cells. The authors reviewed FNAC smears and found the features consistent with histopathological diagnosis i.e. spherical globules of basement membrane material surrounded by tumor cells along with basement membrane material in finger like processes between cell clusters and papillary structures. The cells were hyper chromatic uniform, rounded with little cytoplasm [Figure 2].

Later, to confirm the origin and extent of the growth from the trachea, fiberoptic bronchoscopy was performed which showed a well defined solitary, smooth surfaced, lobulated, gray pink mass at the anterior wall of the trachea in its upper part. The rest of the trachea and bronchial divisions up to segmental level were normal. Biopsy from the tracheal wall mass reveals similar histopathological features. Patient was referred to oncology department where he completed four weeks course of radiotherapy to tumor area with a total dose of 50 Gy. Patient was lost to follow-up thereafter.

## DISCUSSION

ACC arises more commonly in the minor salivary glands and in the seromucinous glands of the upper respiratory tract. Tracheal tumors mostly arise in the lower or upper third, with a tendency to originate at the lateral and posterolateral wall near the junction of the cartilaginous and membranous portions.[9]

Pathologically these tumors characteristically grow into the airway lumen, forming a smooth surfaced, somewhat polypoid tumor; occasionally, growth is circumferential and annular. Submucosal extension, sometimes to a considerable distance from main tumor is not uncommon. Histologically, three patterns are seen; trabecular, cribriform and solid type. The cribriform pattern is most common consisting of uniform cells with relatively little cytoplasm arranged in well-defined nests of variable size. The cells in these nests are separated by well-defined cystic spaces containing a mucinous substance that stains strongly with alcian blue and weakly with Periodic Acid - Schiff (PAS).[10]

Most ACCs are discovered in middle age with no gender predilection. The usual clinical presentation is directly related to the size and location of the tumor within trachea. These tumors may grow to near obstructive level and produce cough, hoarseness, wheezing, dyspnoea, hemoptysis and recurrent pneumonitis.[8] ACC spreads by direct extension, perineural invasion and hematogenous metastasis. Lymphatic spread is uncommon.[10] Direct extension of ACC of the laryngotracheal complex into the thyroid with clinical manifestation as a thyroid nodule has been rarely reported previously.[4–8] In our case also, the tumor has infiltrated between the cartilaginous plates of trachea, muscle and soft tissues of the neck and formed rounded midline swelling in the neck simulating a thyroid tumor without any respiratory symptoms. A case of metastatic ACC of thyroid from an unknown primary presenting as thyroid swelling is also reported.[11]

Extension of an ACC of the larynx and trachea to the thyroid with manifestation as a thyroid nodule is extremely rare. Idowu *et al*,[6] reported two cases of ACC arising in the laryngotracheal complex and involving the thyroid gland by direct extension. In both cases, the initial clinical manifestation was a suspected thyroid mass for which FNAC was performed and papillary carcinoma was cytological diagnosis in one case. Although FNAC is accepted widely as most accurate, sensitive, specific and cost effective diagnostic procedure in the assessment of thyroid nodules, difficulties might arise at times when classical features of ACC are absent or subtle.

Cytological features of ACC are high cellularity with cribriform or trabecular pattern. Cytoplasm is scanty and basaloid. Nucleus is oval to angulate with coarse chromatin, small indistinct nucleoli and no inclusions. The background element may show hyaline globules. These features usually differentiate ACC from other common primary thyroid neoplasms. If enough aspirates are obtained, immunohistochemical analysis is helpful. Primary thyroid neoplasm will express thyroglobulin, thyroid transcription factor-1, and/or calcitonin, while these antibodies will be negative in ACC and most other tumors of extra thyroid origin. In addition, myoepithelial components of ACC express muscle-specific actin and, occasionally S-100, which usually are absent in thyroid neoplasms.[6]

The CT scan is a useful imaging procedure for ACC. It is highly accurate in the assessment of the tumor location, extra luminal extensions, carinal involvement and distant metastasis.[9] With the use of helical CT data sets, multiplannar reconstructions have been shown to facilitate the assessment of patients with airways disease and are known to provide various advantages in terms of image quality. The reformatted images help to assess both the intra and extra luminal growth of the tumor and its longitudinal extent along the tracheal or bronchial wall by allowing the evaluation of extra luminal surrounding tissues.[12] Therefore, helical CT provides precise information about the extent of a tumor, which is important for planning a surgical resection. Unfortunately, this investigation could not be done in our case.

In conclusion, while evaluating a thyroid nodule, one must be vigilant for tumors from extra-thyroidal sites and ACC should be included in the differential diagnosis of midline swelling neck. In patients with unusually aggressive cervical region tumors, a history of previous or distant malignancy, or atypical cytopathological features noted in the aspiration specimen, the possibility of a non-thyroidal neoplasm should be considered that might arise in adjacent structures, as illustrated by our case.
